# A virtual pebble game to ensemble average graph rigidity

**DOI:** 10.1186/s13015-015-0039-3

**Published:** 2015-03-18

**Authors:** Luis C González, Hui Wang, Dennis R Livesay, Donald J Jacobs

**Affiliations:** Department of Bioinformatics and Genomics, University of North Carolina at Charlotte, Charlotte, NC 28223 USA; Department of Physics and Optical Science, University of North Carolina at Charlotte, Charlotte, NC 28223 USA; Center for Biomedical Engineering and Sciences, University of North Carolina at Charlotte, 28223 Charlotte, NC USA; Current address: Facultad de Ingeniería, Universidad Autónoma de Chihuahua, Circuito No. 1, Campus Universitario 2, Chihuahua, Chih, CP 31125 Mexico; Current address: Goldman Sachs, 200 West Street, New York, NY 10282 USA

**Keywords:** Graph rigidity, Pebble game, Constraint topologies, Constraint counting, Mean field approximation, Effective medium, Probability flow, Protein flexibility, Protein stability

## Abstract

**Background:**

The body-bar Pebble Game (PG) algorithm is commonly used to calculate network rigidity properties in proteins and polymeric materials. To account for fluctuating interactions such as hydrogen bonds, an ensemble of constraint topologies are sampled, and average network properties are obtained by averaging PG characterizations. At a simpler level of sophistication, Maxwell constraint counting (MCC) provides a rigorous lower bound for the number of internal degrees of freedom (DOF) within a body-bar network, and it is commonly employed to test if a molecular structure is globally under-constrained or over-constrained. MCC is a mean field approximation (MFA) that ignores spatial fluctuations of distance constraints by replacing the actual molecular structure by an effective medium that has distance constraints globally distributed with perfect uniform density.

**Results:**

The Virtual Pebble Game (VPG) algorithm is a MFA that retains spatial inhomogeneity in the density of constraints on all length scales. Network fluctuations due to distance constraints that may be present or absent based on binary random dynamic variables are suppressed by replacing all possible constraint topology realizations with the probabilities that distance constraints are present. The VPG algorithm is isomorphic to the PG algorithm, where integers for counting “pebbles” placed on vertices or edges in the PG map to real numbers representing the probability to find a pebble. In the VPG, edges are assigned pebble capacities, and pebble movements become a continuous flow of probability within the network. Comparisons between the VPG and average PG results over a test set of proteins and disordered lattices demonstrate the VPG quantitatively estimates the ensemble average PG results well.

**Conclusions:**

The VPG performs about 20% faster than one PG, and it provides a pragmatic alternative to averaging PG rigidity characteristics over an ensemble of constraint topologies. The utility of the VPG falls in between the most accurate but slowest method of ensemble averaging over hundreds to thousands of independent PG runs, and the fastest but least accurate MCC.

## Background

An important characteristic of molecular systems is the number of internal degrees of freedom (DOF) governing conformational variability. In particular, the mechanical properties of a molecular structure depend on the detailed set of interactions that form, which is controlled by chemical composition and thermodynamic conditions [1,2]. To quantify flexibility within a molecular structure, distance constraints between atoms have been used to model covalent bonds and hydrogen bonds as well as other noncovalent interactions [3,4]. As more interactions form, additional distance constraints are added to the network. As new distance constraints are placed in flexible regions, the number of DOF decreases with a corresponding reduction in accessible motion. A distance constraint that removes a DOF from the network is said to be independent. On the other hand, if a new distance constraint is placed in a rigid region, no change in the number of DOF will result, and the distance constraint is said to be redundant. The major goal of rigidity analysis is to identify independent distance constraints. Rigidity algorithms typically represent molecular structure as a graph where edges model distance constraints and vertices model atoms or small molecular rigid clusters.

In two dimensions, the first polynomial algorithm to identify independent edges was proposed by Sugihara [5]. Afterward, several other O(N^2^) algorithms were developed, such as by Imai [6] using a network flow approach, by Gabow and Westermann [7] using matroid sums and by Hendrickson [8] using bipartite matching. Jacobs and Hendrickson [9] improved the efficiency of the bipartite matching algorithm in the context of a pebble game (PG) where a graph is recursively built up one edge at a time. In practice, the PG typically reduces computational cost to O(N) whenever vertex connectivity is representative of molecules or polymeric materials. This gain in efficiency occurs by immediately condensing Laman subgraphs [10] as they are found. Since a Laman subgraph is self-rigid, its connectivity is transformed by triangularization so that any pair of vertices can be reached by at most traversing two edges. In pathological networks, such as the random bond network [11], there are no redundant constraints until the network all at once transitions from floppy to rigid. The worst case performance of O(N^2^) operations occurs in the PG on pathological networks because Laman subgraphs are never detected. Finally, it is worth mentioning that a new hierarchical decomposition method to identify Laman subgraphs was recently developed by Bereg [12].

The PG was initially developed to calculate many properties of graph rigidity for generic two-dimensional networks [13]. Generic rigidity implies that knowing just the constraint topology is sufficient to determine rigidity properties, rather than the specific coordinates of atoms. Rigidity properties include identifying: (1) a set of independent distance constraints; (2) the rigid and flexible regions within a network; and (3) over-constrained regions that have more distance constraints than is needed for the region to be rigid. Extensions to three-dimensions using similar PG algorithms have been made for a limited number of network types [3,14-16], and further generalizations have been made to an entire class of graph rigidity problems involving matroids [17,18]. The basic structure of the PG remains the same in three dimensions when the body-bar model [19] is used. The development of PG algorithms is motivated by applications to polymeric materials and polymers [1], and in particular to predict flexible and rigid regions within proteins [2,3,20]. Motivation for the *virtual pebble game* (VPG) described in this report is to facilitate predicting thermodynamic stability in proteins much more rapidly and with higher precision than that can be achieved by ensemble averaging results from the PG.

Thermodynamic properties of proteins and peptides have been accurately predicted using a distance constraint model (DCM) that regards network rigidity as an underlying mechanical interaction [21-23]. The DCM is a complete equilibrium statistical mechanics model. As such, thermal fluctuations of noncovalent interactions are expressed through an ensemble of accessible constraint topologies, each with its own Boltzmann factor. The free energy landscape is calculated for a protein over a range of macrostates that are specified by the average number of H-bonds and average number of native-like torsion interactions that model good atomic packing. Poor atomic packing is modeled by disordered torsion interactions, which have properties that differ from native-like torsions. Covalent bonding is modeled as fixed distance constraints, while H-bonding and torsion interactions are modeled as fluctuating distance constraints. For each macrostate, the PG must be applied hundreds of times to ascertain a statistically meaningful average probability for the fluctuating distance constraints to be independent or redundant. The results of the PG depend on the number, type and placement of the distance constraints that are present. High and low density of distance constraints appear within protein structure when considering the folded and unfolded states respectively. After the average number of DOF is calculated for each macrostate, the free energy of a protein is subsequently expressed as a function of its global flexibility [22,23].

In practice, the number of PG “plays” on distinct constraint topologies typically add up to a few million for a protein having 150 to 200 residues. We therefore asked if it would be possible to replace averaging over hundreds of PGs per macrostate to obtain accurate information about which constraints are independent or redundant using only one PG on a representative constraint topology. Barring specific details of the DCM that can be found elsewhere [23], the idea is to replace hundreds of PG “plays” for a given macrostate with a single representative PG. In this case, the free energy calculations would be anywhere from 100 to 1000 times faster depending on how much sampling error can be tolerated. With high throughput *in silico* protein design applications in mind, a few-hundred-fold speedup is desirable. This begs the question about how much accuracy must be sacrificed for such gain in speed? In previous works, the DCM was solved using Maxwell constraint counting (MCC), which is viewed in today’s language as a mean field approximation (MFA) that ignores detailed information about constraint topology except the average constraint density [24]. Surprisingly, MCC was sufficient to accurately describe the salient thermodynamic properties of structural transitions for the *β*-hairpin to coil transition [25], the *α*-helix to coil transition [26] and protein folding [27].

The estimate for the number of DOF in a network given by MCC [1,16,24] assumes that a network of *N* atoms is globally flexible with all constraints independent whenever there are less than 3N – 6 constraints present, otherwise the network is globally rigid. Surprisingly, this crude estimate turns out to be markedly accurate when distance constraints are uniformly distributed across an entire network. Of course, molecular systems of interest ubiquitously have fluctuations in local constraint density. Although MCC provides only a mean field estimate for the global response of a system, it does yield a rigorous lower bound to the number of independent DOF within a network. However, MCC enforces a cooperative two state behavior where the native state is globally rigid, and the unfolded state is globally flexible. As such, two-state protein thermodynamics can be captured because the constraint counting errors self-average out in each state, while stark differences between the folded and unfolded states are easy to characterize within MCC.

Unfortunately, all local details regarding which regions in a network are rigid or flexible are lost using MCC. Therefore, a better MFA that counts DOF more accurately in order to retain useful information about identifying flexible and rigid regions provides the impetus for the VPG. The VPG is based on a MFA that follows the spirit of effective medium theory [28]. Specifically, an effective constraint topology is created per macrostate to represent a sub-ensemble of constraint topologies that share specific characteristics of constraints. To calculate the entire free energy landscape, tens of thousands of VPG plays will still be needed in the DCM corresponding to one VPG play per macrostate.

The VPG is designed to calculate ensemble average rigidity properties using a single network by tracing probabilities for pebble placement, rather than moving the pebbles themselves. In this context, distance constraints associated with fluctuating H-bonds are assigned a probability to appear in the network. Probabilities for an interaction to appear are translated into pebble capacities assigned to edges within the graph. A greater pebble capacity for a given type of interaction corresponds to a greater average number of distance constraints. As a consequence of this mapping, pebble rearrangements in the VPG correspond to probability flowing through the network. The key objectives of the VPG are to (1) more accurately calculate the number of DOF in a network compared to the lower bound estimate from MCC, (2) retain network rigidity information similar to the sampled average properties of the PG, and (3) make precise predictions without sampling errors. Although the VPG will cost approximately the same amount of computing time as a single PG, its results are fully deterministic and therefore void of statistical sampling errors. By design, the first and third objectives are ensured by the VPG, where it is as precise as machine precision allows. The usefulness of such a VPG will then depend on how accurate it is compared to the exact ensemble average PG results.

In a previous study [29] the rigid cluster decomposition (RCD) from the VPG was compared to the ensemble-averaged RCD of the PG. Considering a worst case scenario where all H-bond probabilities were treated with equal probability to maximize variance, it was found that the VPG results for the RCD is a faithful representation of the ensemble-average results of the PG. Specifically, it was shown that the RCD results from the VPG provide quantitative insight into protein flexibility that is consistent within statistical error bars of the PG sample averaged over 1000 distinct constraint topologies. Furthermore, fluctuations in the average RCD results were explored in a follow up study [30] by devising a hybrid model where the VPG and the PG were blended together as a linear supposition with a weight factor ranging from 0% to 100% VPG. Comparing speed/accuracy tradeoff, it was determined that there is little to no advantage to ensemble averaging using a PG if the VPG is being used on proteins. In other words, the systematic error that the VPG produces is tolerable in many cases where the size of the statistical error bars of the ensemble averaged PG results is not tolerable.

In this report, all basic elements of the VPG algorithm are described as an isomorphic mapping to a body-bar PG to determine rigidity properties in proteins [15]. Performance and accuracy of the VPG is further characterized by applying it to disordered lattices with a comparison to protein networks. VPG estimates for the number of DOF are compared to the MCC estimates and the ensemble averaged PG results. A heterogeneity index is introduced to quantify local variations in constraint density to investigate the impact on the VPG results when there is greater or lesser degree of spatial variance in the distribution of distance constraints within a network. It is important to emphasize that all the test cases considered in this work, and in previous works, do not employ in anyway the DCM to calculate constraint probabilities. Rather, distinct constraint topologies are generated using spatially invariant probabilities that maximize local fluctuations to purposely consider worst case scenarios. Across a diverse set of test cases presented here, the VPG meets our primary objective to achieve an excellent balance between computational efficiency and accuracy in quantifying network rigidity. Consequently, we expect the VPG will have widespread utility in robustly and rapidly predicting average network rigidity properties similar to its PG counterpart.

## Materials and methods

### Test body-bar networks

A test set comprising of a large number of body-bar networks was created to characterize how accurate the VPG can estimate the number of DOF, as well as the first and second moments of rigid cluster size. These networks vary from protein-like networks to topologies based on disordered lattices that accentuate the role of fluctuating edges. Particular emphasis is given to the results on disordered lattices to benchmark the performance of the VPG under conditions that are far worse than those encountered in protein structure. This is because in proteins there is a covalent bonded backbone chain that supports crosslinking. The backbone chain is made up of quenched distance constraints, meaning they do not fluctuate. Crosslinking distance constraints come from fluctuating H-bonds, and possibly quenched disulfide bonds. Consequently, secondary and tertiary structure of a protein dictates the nature of the network. In disordered lattices, quenched covalent bonds and fluctuating H-bonds are placed in the network at random, allowing the ratio of number of H-bonds to number of covalent bonds to be very different than that found in proteins. The spatial distribution of covalent bonds and H-bonds in disordered lattices is also much more heterogeneous than that found in proteins. In particular, by selecting conditions for disordered lattices that yield a high degree of constraint density fluctuations (described below), the MFA becomes most susceptible to error. As demonstrated below, the VPG provides good estimates for key rigidity properties even on networks that are intrinsically much more heterogeneous than those found in protein structure.

We consider square (d = 2) and cubic (d = 3) lattices with *L* vertices in each dimension, yielding a total of *L*^*d*^ = *N* vertices. Note that the two dimensional case represents a sheet embedded in three dimensional space, which need not be planar, but could ripple and splay like beta sheets do in protein structure. In addition, periodic boundary conditions are used in each dimension for the disordered lattices so that all vertices have 2*d* incident edges. It is important to note that the regularity of the lattice whether in 2 or 3 dimensions is applied to topology, but not to the geometry. Namely, the lattices can be thought of as being distorted. In other words, the atomic geometry of the networks in this study is considered to be generic, and use of periodic boundaries is for convenience, which follows the same rationale given in previous work [13]. Protein structure is modeled based on the coordinates of PDB files, and the structure is assumed to be generic following previous works [3,31].

For constraint networks considered here, each vertex represents a rigid body with 6 DOF, and an edge represents a number of generically placed distance constraints between two rigid bodies. As explained previously [14] the rigid body centered on an atom is a consequence of local covalent bonding to neighboring atoms. This atom to local rigid cluster mapping is based on the molecular conjecture [19] that has recently been proven rigorously [32]. Within this body-bar framework, one of two types of edges can be placed between any pair of vertices: quenched or fluctuating. Quenched edges model covalent bonds and will have probability 1 to be present within the network. Fluctuating edges that model H-bonds, for example, are present in the network with probability, *p*. Assigning a probability to fluctuating constraints reflects the continual process of intramolecular interactions breaking and reforming within the molecular structure, where the interaction is present *p* fraction of the time. Note that for protein structure, the probability for an H-bond to be or not to be present depends on its local environment [23]. Nevertheless, we purposely consider all fluctuating interactions to have the same probability to be present because spatially invariant probabilities are convenient to characterize the VPG across our test set of networks, and, more importantly, for a desired number of H-bonds in the network, employing a uniform probability for all H-bonds corresponds to maximizing constraint density fluctuations across a network. In contrast, non-uniform probabilities can create heterogeneity in the density of distance constraints, but local fluctuation in constraint density is suppressed. Since we wish to benchmark the VPG under worst case conditions, uniform probability is applied to all fluctuating constraints.

**Figure 1:**
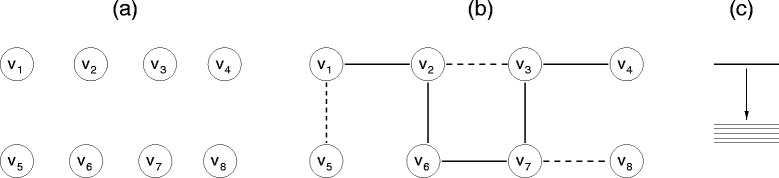
**Creation of a disordered lattice in two dimensions.** At the beginning of the process, **(a)** the network is a set of disconnected vertices. Then **(b)**, edges are placed between nearest neighboring vertices where some are quenched (solid lines) and others fluctuate between being present or not (dashed lines). Missing edges are not shown. **(c)** When an edge is present, it represents 5 bars.

In the disordered lattice networks each vertex has 2*d* nearest neighbor vertices, and each of these pairs of vertices may have an edge that is quenched or fluctuating, or not present at all. An edge is *quenched* with probability *q*_fix_ or the edge is *fluctuating* with probability *q*_fluct_. Neighboring vertices are disconnected with probability (1 − *q*_fix_ − *q*_fluct_). The process of creating a test body-bar network is shown in Figure 1 for a simple case in two dimensions on a square lattice. Initially, we begin with a set of unconnected vertices, each representing a rigid body (Figure 1a). Next, quenched and fluctuating edges are randomly placed between neighboring vertices in the lattice based on probabilities *q*_fix_ and *q*_fluct_ respectively. Specifically, a uniform random number (0 ≤ *x* ≤ 1) is generated for each pair of neighboring vertices. When *x* ≤ *q*_fix_ a quenched edge is placed between the pair of vertices; when *q*_fix_ < *x* ≤ (*q*_fix_ + *q*_fluct_) a fluctuating edge is placed; otherwise no edge is placed.

The probability *q*_fluct_ determines if an edge is going to be part of the set of fluctuating edges (shown as dashed lines in Figure 1b). Once an edge is known to be fluctuating, another probability, *p*, determines if it is present in the network. Over the entire network, the probability, *p*, applies to each fluctuating edge. In the limit that *p* → 0 a fluctuating edge will always be missing, and as *p* → 1 a fluctuating edge will always be present. The probability *p* controls the average amount of cross-linking within the network, and at $p={\scriptscriptstyle \frac{1}{2}}$ the greatest fluctuations occur within an edge. The probabilities {*q*_fix_, *q*_fluct_, *p*} dictate independent random events that can occur for each edge. Subsequently, a Monte Carlo process generates a sample of networks of specific character. The pebble capacity of an edge is set equal to the number of distance constraints that connect the incident vertices of the edge. Following earlier works that model covalent bonds and H-bonds using 5 distance constraints [3,15,16,31], we assign a pebble capacity of 5 to an edge that is present, 0 to an edge that is missing, and 5*p* to reflect an average pebble capacity for a fluctuating H-bond.

One to one comparisons between the PG and VPG are done on networks where the placement of quenched and fluctuating edges are identical. For a network with N_*f*_ fluctuating edges, an ensemble consisting of ${2}^{N_f}$ different realizations must be generated to obtain an exact average for any quantity of interest, such as the number of independent constraints. In the example shown in Figure 1b, the edges *v*_1_ − *v*_2_, *v*_2_ − *v*_6_, *v*_6_ − *v*_7_, *v*_3_ − *v*_7_ and *v*_3_ − *v*_4_ are quenched describing covalent bonding, while the edges *v*_1_ − *v*_5_, *v*_2_ − *v*_3_ and *v*_7_ − *v*_8_ describe fluctuating H-bonds. Adjacent vertices not connected by an edge represent “missing” interactions (e.g. *v*_5_ − *v*_6_). When an edge is present, it represents 5 distance constraints generically placed between two rigid-bodies that can be bundled together as shown in Figure 1c. Hereafter, a distance constraint is referred to as a bar. It is worth pointing out that the PG employs a multigraph because each bar is treated as an edge, and multiple edges can join a pair of vertices to facilitate exact integer counting. In contrast, integer counting is irrelevant in the VPG because a constraint capacity is assigned to each edge. For example, a capacity of 5 represents 5 bars bundled together. Hence, the VPG is not a multigraph because a single edge represents the average number of bars present via its pebble capacity.

**Figure 2:**
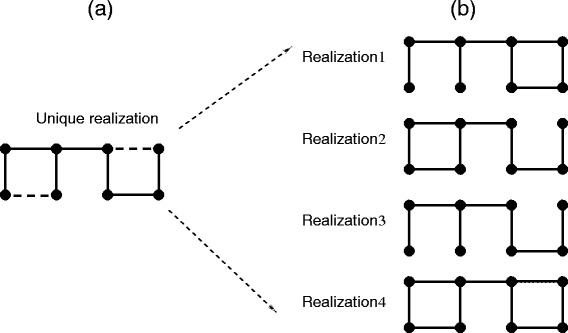
**A single unique representative VPG network. (a)** Shown as a dashed line the VPG assigns a pebble capacity to each fluctuating edge equal to its average number of bars present. **(b)** An exhaustive set of possible PG realizations. In each realization, the fluctuating edge is either missing (no line) or is present (solid line). Missing and quenched edges are shared commonly between the VPG and each PG realization.

Once the number and placement of quenched and fluctuating edges are specified, a few hundred samples is usually sufficient to obtain PG averages with acceptable statistical error bars. To be clear, note that for each sample an independent uniform random number (0 ≤ *x* ≤ 1) is generated for each fluctuating edge. When *x* ≤ *p*, the fluctuating edge is assigned 5 bars, otherwise no bars are assigned. Within the VPG, fluctuating edges are assigned a pebble capacity of 5*p* because on average five bars are present with probability *p*, and zero bars are present with probability 1 − *p*. Thus, a fluctuating edge in the VPG becomes a quenched edge that removes 5*p* pebbles. An example of how an ensemble of PG networks with two fluctuating edges map to a representative network is shown in Figure 2. In this case the ensemble of PG networks consist of 4 distinct constraint topologies.

### Constraint counting on lattices

Within MCC, all distance constraints are assumed to be independent within a flexible structure until a rigidity transition takes place where the network becomes globally rigid. Formally, MCC is a MFA that neglects fluctuations in the density of constraints throughout the structure. For the body-bar disordered lattices considered here, Equation 1 is the MCC lower bound estimate for the mean number of internal DOF over an ensemble of networks having different placements of quenched and fluctuating edges.

1 $$ {F}_{\mathrm{MCC}}= \max \left[\kern0.1em 6{L}^d-5d\kern0.1em {L}^d\kern0.1em \left({q}_{\mathrm{fix}}+p\kern0.1em {q}_{\mathrm{fluct}}\right)-6, \kern0.24em 0\kern0.1em \right] $$

Operationally Equation 1 subtracts from the maximum number of DOF in the system (i.e. 6*L*^*d*^) the average number of constraints and 6 trivial DOF related to rigid body translations and rotations. Since we employ periodic boundary conditions, the number of incident edges per vertex in a regular hyper-cubic lattice is 2*d*. Note that because each edge connects two vertices, dividing by 2 prevents double counting. Hence, there are *dL*^*d*^ maximum edges in the network, and with 5 bars per edge the maximum number of distance constraints that can appear is 5 *dL*^*d*^. At the point of perfect balance (i.e. *F*_MCC_ = 0) in the number of DOF and constraints, a rigidity threshold is defined where the character of the network transitions from globally flexible with no local rigid parts to globally rigid with no flexible parts as new constraints are added. Note that for heterogeneous rigid and flexible regions to appear there must be constraint density fluctuations.

To check the limits of Equation 1, when there are no distance constraints at all (i.e. *q*_fix_ = *q*_fluct_ = 0) the total number of internal DOF is correctly calculated to be 6(*L*^*d*^ − 1). As *q*_fix_ or the product of probabilities, *p q*_fluct_ increases, more distance constraints are added to the network, which leads to a decrease in internal DOF. Here, we consider either a square lattice representing a sheet (*d* = 2) embedded in three-dimensional space, or a cubic lattice (*d* = 3). Given that every edge is tied to 5 bars, each vertex has a maximum of 20 and 30 incident bars for *d* = 2 and *d* = 3, respectively. Since these full lattices possess a high density of constraints at each vertex, many constraints will be redundant long before MCC predicts the rigidity threshold to take place corresponding to when *F*_MCC_ → 0^+^. Note that without the max() function in Equation 1, MCC gives a negative estimate for the number of internal DOF, but this number reflects the number of redundant constraints. An important characteristic of Equation 1 is that the number of available DOF within the network is an extensive quantity, being proportional to the number of vertices within the network.

### Description of the body-bar VPG algorithm

For generic body-bar networks, exact constraint counting using an integer algorithm was previously implemented as a pebble game (PG) [15], which serves here as the starting point for the VPG. In the PG, each DOF is represented by one pebble and each distance constraint is represented by one edge. Since multiple distance constraints can connect the same pair of rigid bodies, the body-bar PG is based on a multigraph where multiple edges can connect to a given pair of vertices. Each vertex has 6 free pebbles to account for 3 translational and 3 rotational DOF. The PG builds up the network from a set of isolated vertices by placing one edge at a time in a recursive fashion. Operationally, pebbles may be rearranged within the network to cover added edges like explained for the 2D PG [9]. The body-bar PG algorithm has been proved based on the matroidal property of sparse graphs [17,18,32].

A redundant edge is identified when seven free pebbles cannot be accumulated on its two incident vertices. To test if an edge connecting vertex *v*_*a*_ to vertex *v*_*b*_ is independent, 6 free pebbles are first collected on vertex *v*_*a*_. Note that six free pebbles can always be collected on any vertex. The next step is to hold fix the 6 pebbles to vertex *v*_*a*_ while attempting to collect a seventh free pebble on vertex *v*_*b*_. If the seventh pebble is found, the edge is independent and the seventh pebble is used to cover the edge. As independent edges are covered, the number of free pebbles on vertices decreases. If on the other hand, a seventh pebble could not be found, the edge is redundant and cannot be covered by a pebble. While the order of edge placements affects which edge is identified as redundant or independent, the total number of independent constraints and DOF within the network do not depend on this ordering. Furthermore, the rigid cluster decomposition is unique, and, all rigid clusters comprise of contiguous sets of nearest neighbor vertices.

The VPG inherits all key properties of the PG, where operationally the VPG is isomorphic to the PG. The main difference is that the number of pebbles that are free on a vertex or that cover an edge is described by a real number instead of an integer. As a consequence of this distinction, the multigraph nature of the PG is mapped onto a graph with only one edge between a pair of vertices, but each edge is assigned a capacity. Whereas in the PG *n*-distinct edges are needed to represent *n* bars, in the VPG, one edge is assigned a capacity of *n* pebbles because the bars are bundled together. For example, for 5 bars the edge has a capacity of 5 as shown in Figure 1c. Before generalizing to real numbers it is worth noting that assignment of integer capacities to edges is a special case of the VPG that results in a faster PG implementation than the original multigraph PG implementation [15]. Consolidation of information makes moving more than one pebble at a time possible, which reduces the average number of pebble searches and average pebble search length. Roughly, the VPG runs about 20% faster than the PG.

The main difference between the VPG and PG is conceptual (not algorithmic) because the MFA suppresses fluctuations in the number of bars that are present within edges. The term “virtual” is used because pebbles are no longer discrete entities, but rather represent an average amount of pebbles that flow within an effective constraint network where edge capacities rate limit the flow of pebbles through an edge. Edge capacities will generally be spatially inhomogeneous. In all other ways, operations involving pebble rearrangements in the VPG are identical to the PG. In particular, the directional nature of pebbles covering edges in the VPG preserves the critical bipartite matching aspect of the PG algorithm, so that tracing through covered edges provides a *viable path* from vertex *v*_*a*_ to *v*_*b*_ only when the edge is covered by pebbles from *v*_*a*_ to *v*_*b*_. A backflow from vertex *v*_*b*_ to vertex *v*_*a*_ is not guaranteed. The flow from *v*_*b*_ to *v*_*a*_ requires the edge to be covered by pebbles from vertex *v*_*b*_. The forward flow rates and backflow rates depend on the amount of pebbles that cover an edge from two different directions, and in general they are both present at the same time, but not equal. Local correlations in pebble flow paths in the VPG have the exact same restrictions as the PG.

In the VPG, the amount of pebbles that cover an edge represent all possible PG runs simultaneously. Hence, the pebble capacity of an edge in the VPG is set to the average pebble capacity across the ensemble of all possible constraint networks. Because fluctuating edges are tied to independent and identically distributed random variables, this average is 5*p*. Employing the pebble rearrangement rules of the PG enforces local conservation of pebble flow through edges and vertices. In particular, the amount of pebbles covering an edge remains constant as pebbles flow through the network. An important point is that the bidirectional nature of the PG is preserved in the VPG. As such, the total number of pebbles covering an edge consists of adding the amount of pebbles covering an edge from both directions. Hence, the search for pebbles in this directed graph resembles a network flow problem [33,34], where edge capacities determine the maximal flow of pebbles through the network.

To describe the VPG algorithm consider a network consisting of vertices {*v*_*n*_}, *n* = 1, 2, … *N*, with a list of edges {*e*_*m*_}, *m* = 1, 2, … *M*. Let the capacity for the *m*-th edge be denoted as *c*_*m*_. The VPG follows the following procedures and operations:

1. Initialize the graph with a set of isolated vertices {*v*_*n*_}, with six pebbes assigned to each vertex.
2. From the list of edges {*e*_*m*_}, insert edge *e*_*k*_ with capacity *c*_*k*_ into the graph. Let *v*_*i*_ and *v*_*j*_ be the two incident vertices for edge *e*_*k*_.
3. Collect 6 pebbles for vertex *v*_*i*_ by performing a breadth first search.
4. Flag vertex *v*_*i*_ as visited, and then attempt to collect *c*_*k*_ pebbles for vertex *v*_*j*_ by performing a breadth first search while holding the 6 free pebbles on *v*_*i*_ in place. If some, but not all *c*_*k*_ pebbles are collected, then continually try to collect more pebbles by performing breadth first searches repetitively until there are enough free pebbles on *v*_*j*_ to cover edge *e*_*k*_. On the other hand, upon the first time no free pebbles can be collected on vertex *v*_*j*_ indicates a failed pebble search.
5. If *c*_*k*_ or more pebbles are collected on vertex *v*_*j*_; cover edge *e*_*k*_ with *c*_*k*_ pebbles, and declare this edge as independent. Otherwise, if less than *c*_*k*_ pebbles are collected on vertex *v*_*j*_; all the visited vertices within the failed search are condensed into a single vertex, which is assigned zero free pebbles. Go to step 2 until all edges in the edge list, {*e*_*m*_}, has been placed.
6. End of VPG.

**Figure 3:**
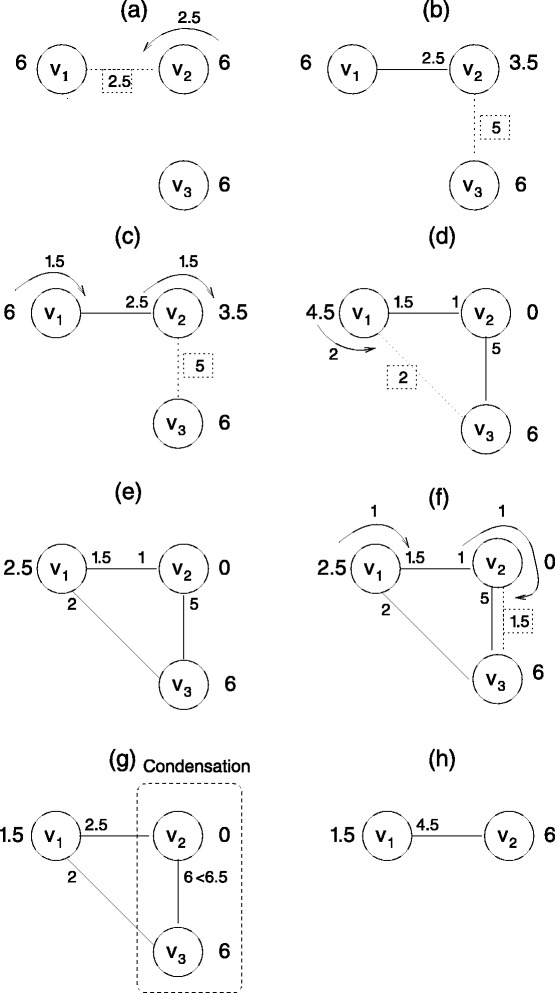
**Illustration of how to play the VPG on a network with 3 vertices.** A dashed line denotes the edge that is being added at the current step, whose capacity is indicated in a dashed box. Appearing at the ends of an edge is the number of pebbles consumed from the corresponding vertex used to cover the edge. The operations that rearrange pebbles are described as a sequence of labeled steps. Numbers listed in dashed boxes refer to pebble capacities of an edge that is to be placed, but not yet placed in the network until the required number of free pebbles can be collected. **(a-b)** Each vertex is initially assigned 6 pebbles, and an edge of capacity 2.5 is added to the graph. **(c)** Vertex *v*_2_ has 3.5 free pebbles and cannot fully cover the new edge between *v*_2_ and *v*_3_ (which requires 5 pebbles). A pebble search is carried out and 1.5 pebbles are backtracked through the edge between *v*_1_ and *v*_2_. **(d-e)** Vertex *v*_1_ has enough free pebbles to cover the newly added edge. **(f)** Adding an additional edge between *v*_2_ and *v*_3_, the two edges (of capacity 5 and 1.5) combine, yielding a partially covered edge with capacity 6.5. Of course physically, the greatest possible covering is 6. As such, only six pebbles can cover the edge. **(g)** Because the edge between *v*_2_ and *v*_3_ cannot be fully covered, the attempted pebble search fails, which leads to the condensation of *v*_2_ and *v*_3_ into a single vertex denoted as *v*_2_. **(h)** Edges *v*_1_ − *v*_2_ and *v*_1_ − *v*_3_ in step **(g)** are combined into one edge *v*_1_ − *v*_2_.

An example shown in Figure 3 illustrates VPG operations. The edge list used to construct the graph that include specifying the pebble capacity between pairs of vertices, is given as *v*_1_ − 2.5 − *v*_2_, *v*_2_ − 5.0 − *v*_3_, *v*_1_ − 2.0 − *v*_3_, *v*_2_ − 1.5 − *v*_3_. The operations that are both illustrated and described in Figure 3 include: (1) pebble rearrangements between vertex and edge (Figure 3a); (2) pebble backtracking (Figure 3c); and (3) condensation (Figure 3g). A collection of key VPG pseudo-codes is provided in the Appendix. While different implementations are possible to determine when two real numbers are equal on a digital computer, usually two real numbers are considered equal if they are within some tolerance. We break 1 pebble into 1 billion parts, and perform exact integer comparisons among integer components. This approach yields a precision that is good to 1 part in 10^9^, while maintaining exact counting.

The essential step in the VPG is to collect *c*_*k*_ pebbles on vertex *v*_*j*_ by employing a pebble search multiple times. How the pebble search is implemented does not matter provided it is exhaustive. When a failed search occurs, all the vertices visited become part of a minimally rigid object in the body-bar representation [15]. We call these failed searches “Laman subgraphs” because of the analogous correspondence with the original 2D pebble game [9]. The vertices that comprise the Laman subgraph are subsequently condensed into a representative vertex having 6 pebbles. The process used in the VPG is identical to that used in the corresponding body-bar PG. This condensation procedure dramatically reduces the effective size of the network as Laman subgraphs are detected, resulting in a typical performance from *O*(*N*^2^) to *O*(*N*).

## Results and discussion

**Figure 4:**
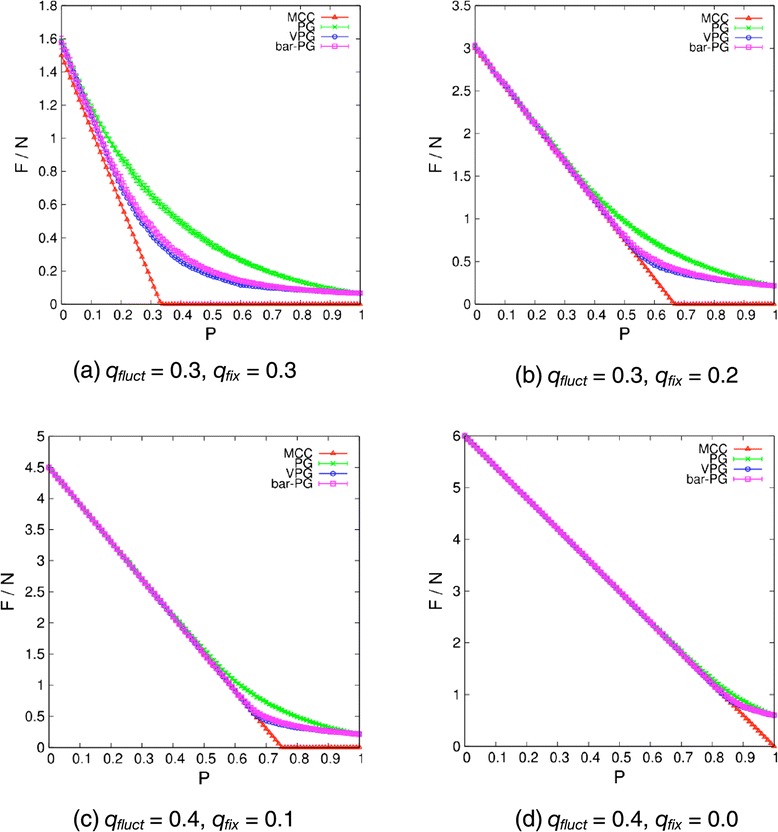
**Comparisons for the average number of internal DOF per vertex.** The average number of internal DOF per vertex within the network for *L* = 20 are plotted as a function of fluctuating edge probability, *p*, based on Maxwell counting (red triangle), ensemble averaged PG (green cross), VPG (blue circle), and ensemble averaged bar-PG (purple square). The straight line is the lower bound estimate given by MCC. The VPG falls in between the PG and MCC results, which gives a much better approximation to the PG than MCC. The maximum error between the PG and VPG occurs at values of *p* close to the Maxwell rigidity transition.

It is convenient to divide the average number of internal DOF (denoted by *F*) by the number of vertices in a network to obtain an intensive quantity. The number of internal DOF per vertex as a function of the fluctuating edge probability, *p*, is shown in Figure 4 at four exemplar values of *q*_fix_ and *q*_fluct_. The examples shown are representative difficult cases, as opposed to being trivially the same as *q*_fix_ → 1. For each of these cases the results from MCC, VPG, and an ensemble-averaged PG on two types of networks are compared. The first type of network that was described above is cooperative because either all or none of 5 bars are present within fluctuating edges. A second type of network is considered where the 5 bars within a fluctuating edge are non-cooperative by modeling each bar as an independent and identically distributed random variable. We label the ensemble-average PG results for the cooperative networks PG, and bar-PG for the non-cooperative networks. Juxtaposition of the PG, bar-PG and VPG is interesting because as a MFA the VPG cannot distinguish between these two types of networks. The random character of a non-cooperative network will be explained in detail below.

The results for all four methods being compared in Figure 4 are based on networks with 8000 vertices (*L* = 20, *d* = 3) that share the exact same set of quenched, fluctuating and missing edges. For the two ensemble-based PG cases, averaging is performed over 100 randomly generated realizations for each value of *p*. Like the original PG, the VPG results for the number of internal DOF within a network are independent of the order that edges are placed, which was extensively verified in this work. In particular, the VPG was run on the same network tens of thousands of times, but with the order of constraint placements randomly reshuffled each time. Despite very different paths in building up the network, identical results are always found. Moreover, an edge with a certain capacity was divided into a large number of parts in unequal portions, and these different parts were placed in random order into the network. Over tens of millions of checks on different ways to reach the same final network, it is found that identical results are obtained regardless of the path taken to build up the network.

Interestingly, for PG it is possible to build a network up in constraint density through a bootstrapping method by first placing a certain number of constraints at random, and then adding more constraints. For example, if a disordered lattice has 10000 fluctuating edges, 10 edges can be placed in the network at random, to yield *p* = 0.001 as the fraction of fluctuating edges present. Then by adding 10 more random edges in succession, a sweep from *p* = 0 to *p* = 1 is made where all *p* values are calculated in increments of 0.001 where the next PG starts from the previous network results. As such, the PG does not restart from the no-constraint case each time. While this procedure is useful for the PG, constraint placement correlations between different networks do appear. This same procedure can be applied to the VPG, where it starts at *p* = 0, and then all fluctuating edge capacities are incremented by *Δp* = 0.001 until *p* = 1 is reached. The total time of calculations for PG versus VPG is comparable, since the VPG at the next increment also starts from the previous case. However, in contrast to PG, no induced correlations appear because each state is independent of the path taken to build up the network.

As expected, MCC overestimates the minimum number of constraints needed for the network to become rigid. When invoking MCC, the rigidity threshold is defined as the lowest value of *p* for which *F*_MCC_ = 0. When *F*_MCC_ > 0 the network is flexible, and when *F*_MCC_ = 0 the network is rigid. In the PG, a network will generally have many localized rigid and flexible regions. Spatial inhomogeneity of flexible and rigid regions is caused by variations in distance constraint density across the network. Due to a mixture of rigid and flexible regions at the rigidity threshold, the true number of internal DOF will not be zero at the rigidity transition. It is also worth noting that the rigidity threshold as determined by the PG is usually below the MCC prediction, but this is not necessary as demonstrated by many examples. The VPG results are much closer to PG calculations than MCC, especially above the rigidity transition in the rigid phase. Below the rigidity transition, the VPG estimate of *F* is underestimated compared to the PG results across intermediate values of *p*.

Relative to MCC, the improved accuracy of the VPG occurs because it applies a MFA locally at the edge level. The VPG averages the constraint density at the edge level, and it does distinguish between quenched, missing and fluctuating constraints. Keep in mind that at the edge level the VPG replaces the random fluctuations of bars with its average value without regard to the underlying random process. For the test networks described above, a fluctuating edge is associated with either five or zero bars with probability, *p* or 1 − *p* respectively. As such, quenched, fluctuating and missing edges are assigned a pebble capacity of 5, 5*p* and 0 respectively.

It is interesting to note that due to the cooperative nature of a fluctuating edge as having either all 5 bars or no bars, fluctuations in the number of bars is maximal in these networks. Because the VPG is a MFA, it is expected that the VPG results will be in better agreement with ensemble averaged PG results if the constraint density fluctuations at the edge level were less. Although the nature of chemical bonding in molecular networks imposes a high level of cooperativity, applications of the VPG can extend beyond molecular networks. In the bar-PG, the five bars within a fluctuating edge are placed independent of each other, each with probability *p*. Thus a fluctuating edge is allowed to have {0, 1, 2, 3, 4, 5} bars, where each bar has an independent probability of *p* to be present and 1 − *p* to be absent. The average number of bars that will be present within an edge is given as 5*p*. Therefore, the two types of networks share the same average property, but edge fluctuations are greatly reduced in the bar-PG relative to PG. The VPG is based on the average pebble capacity of an edge, which is identical for the PG and bar-PG. Quite spectacularly, Figure 4 demonstrates that the VPG results are markedly close to the bar-PG results simply by suppressing fluctuations within edges, regardless of the large-scale heterogeneity of where constraints are distributed.

It would be nice if the VPG could be used to provide a lower bound estimate to the number of internal DOF to the PG, but such a lower bound estimate is obviously impossible. The VPG estimates average behavior of the PG, and one can always find a realization that has all fluctuating edges with all its bars present, and hence the VPG would have a greater number of internal DOF compared to that particular realization. However, it is possible that the VPG provides a rigorous lower bound estimate to the exact ensemble average number of internal DOF. We observe that the estimate for the average number of internal DOF from the VPG is always lower than the sampled PG and sampled bar-PG averages, suggesting such a lower bound may exist. Attempting to prove this type of rigorous result on the VPG opens up new research directions in the field of combinatorial optimization.

**Figure 5:**
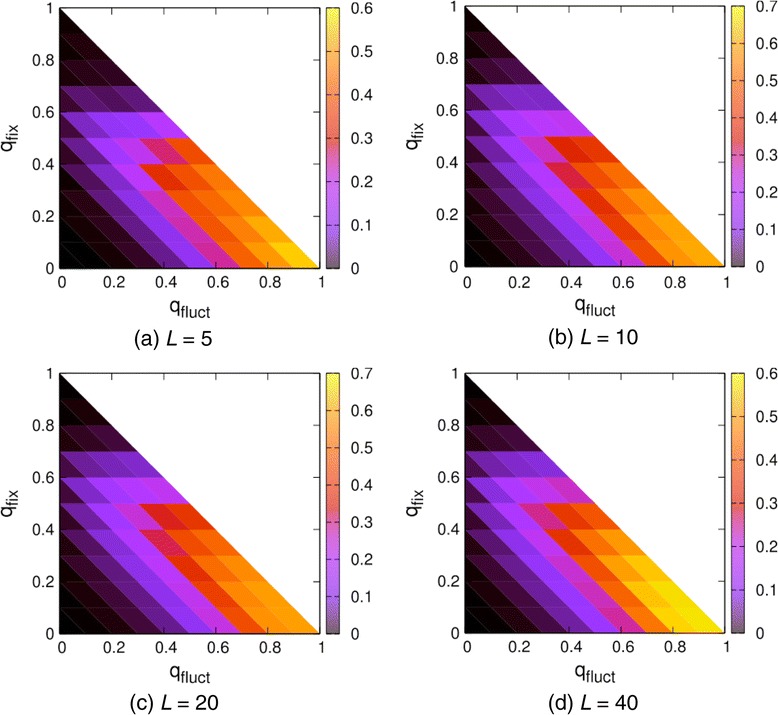
**Contour plots showing the maximum errors in the internal DOF per vertex across four different system sizes.**

For a comprehensive comparison between the VPG and PG results, contour plots given in Figure 5 show the maximum error of the internal DOF per vertex, which is given by

2 $$ \frac{\Delta F}{N}=\frac{ \max \left[{F}_{\mathrm{pg}}(p)-{F}_{\mathrm{vpg}}(p)\right]}{N} $$

The maximum error in Equation 2 is determined by scanning *p* in the range (0*,* 1). The value of *p* at which the maximum occurs varies for different *q*_fix_ and *q*_fluct_ values. The PG algorithm was run on 200 realizations for each set of probabilities {*q*_fix_, *q*_fluct_, *p*}. As expected, the maximum error occurs when *q*_fluct_ = 1. The maximum error of *ΔF*/*N* ≈ 0.6 is found in the worst case, corresponding to a 10% relative error in the internal DOF count considering there are 6 DOF per vertex. However, the maximum error depends on the details of the network topology. Based on the discussions above, MCC is more accurate when the density of constraints in the network is more uniform.

To better understand the computational accuracy of the VPG in relation to the heterogeneous character of the network topology, a quantitative measure, the *heterogeneity index*, is introduced. We define the heterogeneity index, *h*_I_, as the standard deviation of the coordination number (degree of a vertex) across all vertices throughout a plucked network without any dangling ends. The plucked network is obtained by the following procedure. After a network is generated, any vertex with degree one is deleted. That is, any vertex in the network with a coordination number of one is a dangling end, and it is plucked, which will change some degree 2 vertices to degree 1 by creating new dangling ends. This process is repeated until no more vertices have a degree of one, and thus no more vertices can be removed by plucking. After stripping off all dangling ends, the remaining network directly supports percolation of rigidity across the network. Plucked networks were used previously to obtain universal behavior of rigidity lost in protein unfolding as shown in [31]. Based on plucked networks, the formula for the heterogeneity index is applied, given by

3 $$ {h}_I=\sqrt{\frac{1}{N-1}{\displaystyle \sum_{j=1}^N{\left({d}_j-\overline{d}\right)}^2}} $$

where *d*_*j*_ is the degree of vertex *v*_*j*_, and $\overline{d}$ is the average degree of vertices within the plucked network.

Simply put, the heterogeneity index is the standard deviation in vertex connectivity in a plucked network. Initially Equation 3 was applied to the original network containing dangling ends. However, DOF from dangling ends are always counted in perfect agreement between the VPG and average PG because dangling ends never create redundant constraints. It is worth pointing out that the maximum error is calculated to be the same on both the original and plucked networks. However, plucked networks emphasize constraint fluctuations responsible for errors caused by the MFA. We calculated *h*_I_ numerically for each randomly generated network, meaning a fluctuating edge is present or not based on a specific realization (sample).

**Figure 6:**
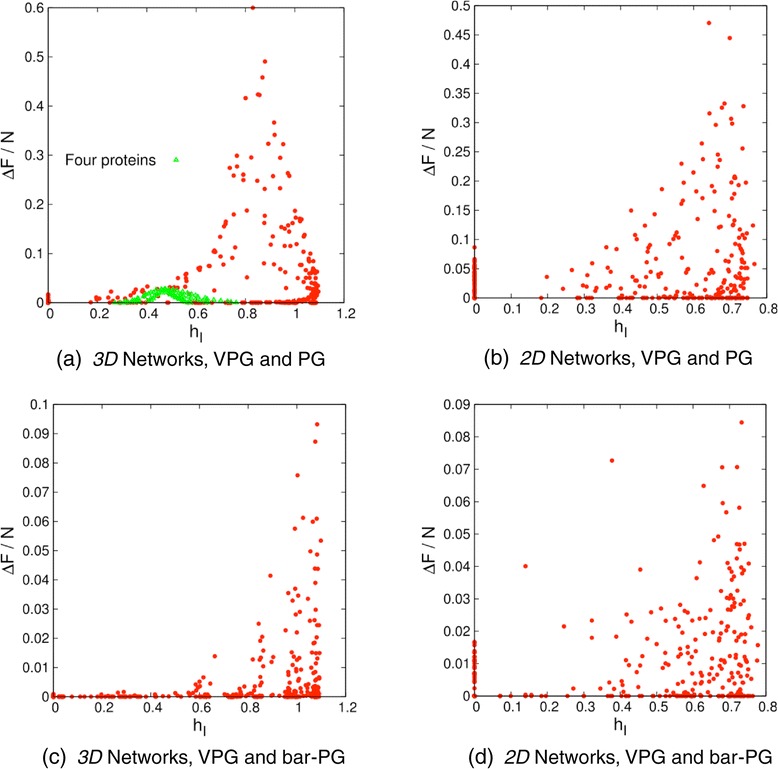
**Maximum difference of internal DOF per vertex between ensemble averaged PG and VPG versus heterogeneity index.** The data is collected for different combinations of *q*_fix_ and *q*_fluct_. Panels **(a)** and **(b)** correspond, respectively, to 3D and 2D lattices with *L* = 20. Panels **(c)** and **(d)** show errors that are much smaller between the VPG and the bar-PG. In addition, in panel **(a)** shows in green symbols the results obtained for 4 different proteins as explained in the text.

The hypothesis that the VPG would increase in accuracy as *h*_I_ decreases proved wrong. Instead, Figure 6 reveals a complicated situation. Because stratifying the data does not lead to any simplifications, the plots in Figure 6 combine all data for various cases of probabilities {*q*_fix_, *q*_fluct_, *p*}. A prevalent feature that stands out is that the converse that a large *h*_I_ implies low accuracy is not true. This is because as *p* → 1 the VPG results are essentially exact, yet *h*_I_ can be large because of the particular choice of *q*_fix_ and *q*_fluct_. Thus, a monotonic trend between *h*_I_ and accuracy of the VPG is not possible. Although errors in the count of internal DOF between the PG and VPG are typically smaller when *h*_I_ is smaller, errors at *h*_I_ = 0 occur. Errors at *h*_I_ = 0 were traced to situations when both *q*_fix_ and *q*_fluct_ are small, and when *p* is close to 1. In this case, it is rare to find rings in the network, but small rings do appear. Deviations between VPG and PG occur in a normal way, yet *h*_I_ = 0 because the plucking process eliminates everything in the network except isolated rings, where all vertices have a degree of connectivity of 2, and hence *h*_I_ = 0. Surprisingly, similar behavior is found in 2D and 3D networks as shown in Figure 6a and b respectively. The rationale for constructing a network with 2D topology is because when applying MFA, fluctuations are generally less important when nearest neighbor connectivity is greater. Contrary to expectations, the VPG was slightly more accurate for 2D networks. Thus, VPG accuracy holds up in 2D topologies markedly well. Interestingly, the largest errors occur at a characteristic value of *h*_I_ (≈0.83 for d = 3, and ≈ 0.64 for d = 2), which demonstrates heterogeneity in edge connectivity does influence the VPG, but in a much less obvious way than that for MCC.

The above analysis reveals that VPG accuracy is not simply related to local homogeneity. However, it was shown above that VPG accuracy improves by suppressing fluctuations in the number of bars using bar-PG. To demonstrate this further, the maximum error of internal DOF between VPG and bar-PG is again plotted against the heterogeneity index for the 3D and 2D cases shown in Figure 6c and d. Similar qualitative dependence of maximum error on *h*_I_ is found compared to the maximum error between VPG and PG. Most significantly, the scale of the errors is decreased by almost an order of magnitude. Also significant is that many of the errors for large values of *h*_I_ for the bar-PG is about the same as for PG. It also appears that for some small values of *h*_I_ the error in the bar-PG are higher. However, this is because the data cannot be mapped one to one, and the *h*_I_ values for non-cooperative fluctuations shift to lower values, since the all or no bars in a fluctuating edge increases the heterogeneity index.

In most regimes of the parameter space {*q*_fix_, *q*_fluct_, *p*} for disordered lattices, the VPG provides markedly accurate estimates for the number of internal DOF. Moreover, maximum errors shown in Figure 6 always result in a relative error of less than 10%, which is sufficiently accurate for a broad range of applications. The disordered lattices were analyzed specifically because they exacerbate spatial fluctuations, which tend to undermine the MFA. Importantly, the character of protein structure suppresses fluctuations. To highlight this point, the maximum error between VPG and PG versus *h*_I_ is plotted in Figure 6a over a dataset of four proteins that are non-redundant at the SCOP [35] family level. We selected a scorpion toxin (pdbid = 1AHO) [36], the biomedical relevant oncogene MTCP-1 (pdbid = 1A1X) [37], the FLAP endonuclease from *M. jannashii* (pdbid =1A76) [38] and a DNA transcription regulator (pdbid = 3COQ) [39]. In proteins, covalent bonds are represented by quenched edges, and H-bonds are represented by fluctuating edges. Following previous work [29], the probability, *p*, for a H-bond to be present is the same for all H-bonds.

**Figure 7:**
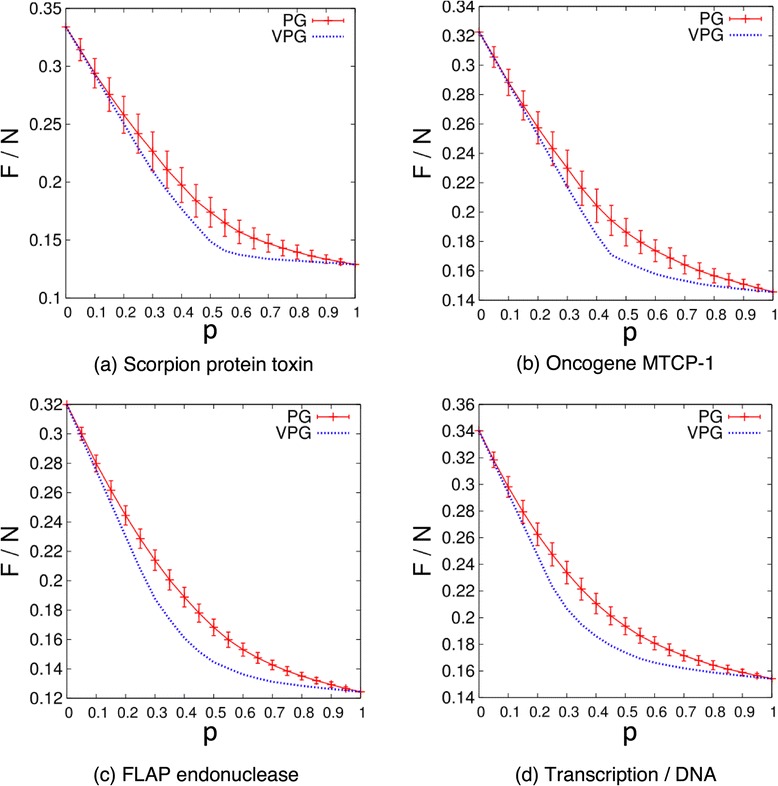
**Comparing the ensemble averaged PG to the VPG in proteins.** The average number of internal DOF per vertex for four proteins is plotted as a function of fluctuating edge probability, *p*, based on ensemble averaged PG and VPG. The standard deviation for PG is shown for comparison. The VPG provides a good approximation for the PG results.

It is clear from Figure 6a that protein networks exhibit a more uniform coordination number than disordered lattices. More important, the maximum errors in estimating the number of internal DOF drop by more than an order of magnitude compared to disordered lattices. As such, the VPG is an excellent approximation to the PG in applications to proteins, as demonstrated in Figure 7 for the four representative proteins showing *ΔF*/*N* as a function of the fluctuating edge probability, *p*. In proteins, we also average over 100 PG samples to get ensemble averages. Notice that the VPG has infinite precision (zero error bars) compared to the sampling error bars seen in Figure 7. While the VPG produces systematic error in the number of internal DOF that underestimates the exact results, it having infinite precision can be more important for applications involving comparative analysis where relative error can cancel out.

**Figure 8:**
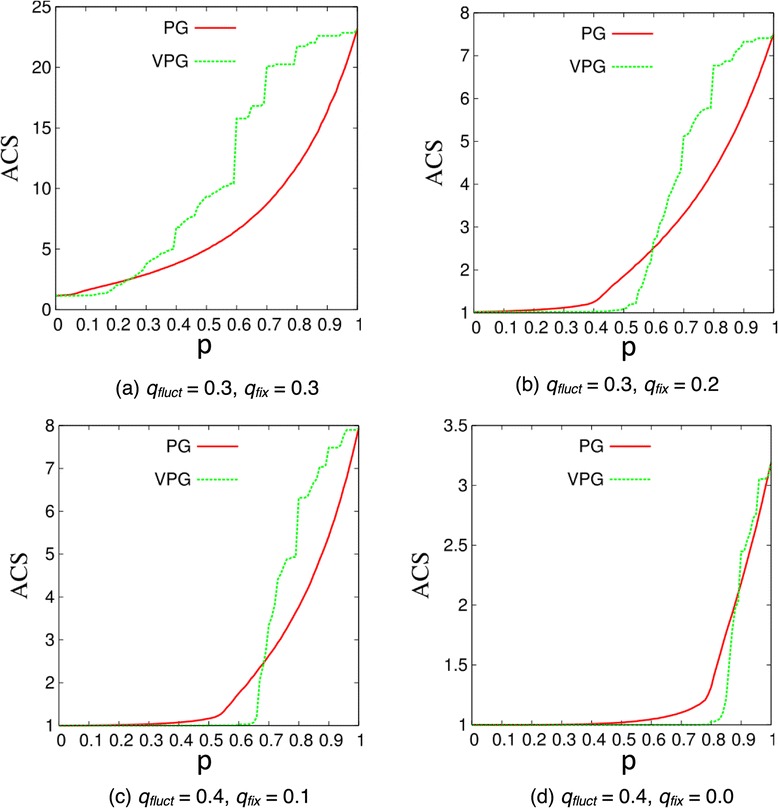
**Average cluster size (ACS) for four exemplar lattice cases as a function of the fluctuating edge probability,** ***p*** **, based on ensemble averaged PG and VPG.**

**Figure 9:**
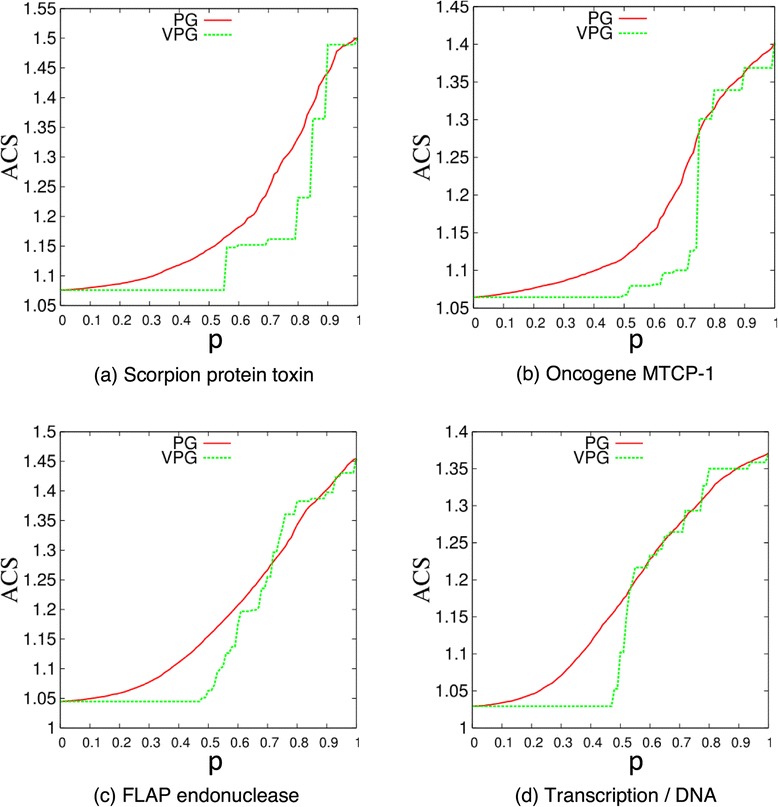
**Average cluster size (ACS) for four protein networks as a function of the fluctuating edge probability,** ***p*** **, based on ensemble averaged PG and VPG.**

We extend the algorithm comparison to the identification of rigid clusters. After all distance constraints are placed within the network, both the PG and VPG determine the number of DOF that are represented as free pebbles on vertices throughout the graph. Regions with an excess number of pebbles are flexible in contrast to a set of vertices that cannot share more than 6 pebbles, and hence form a rigid cluster. More specifically, for two vertices to belong to the same rigid cluster, there must be a maximum of six DOF between them. In order to identify rigid clusters, the counting of pebbles is carried out between pairs of vertices. The method to identify all rigid clusters for the VPG is exactly the same as that of the PG [15]. In *O*(*N*) operations on a graph with N vertices, each vertex is assigned to a unique rigid cluster. When all the vertices are assigned to a rigid cluster it is possible to calculate the average number of vertices per cluster, this quantity called average cluster size (ACS) [29] is calculated by

4 $$ ACS=\frac{1}{N_c}{\displaystyle \sum_{c=1}^{N_c}{N}_V(c)} $$

where *N*_*c*_ is the number of rigid clusters in the network, and *N*_*V*_(*c*) is the number of vertices belonging to the c-th rigid cluster. The VPG is compared to the PG with respect to the ACS across the fluctuating edge probability, *p*, for four exemplar disordered-lattices shown in Figure 8, and for the four proteins shown in Figure 9. When *p* is small, most of the fluctuating edges are missing, and most of the rigid clusters consist of very few vertices. When *p* increases, the sizes of the rigid clusters increase since more of the fluctuating edges will be present, and then crosslinking will merge rigid clusters into larger rigid clusters. This increase in average cluster size will reach a maximal point when all the fluctuating edges are present in the network as *p* → 1. Clearly, the VPG provides ACS estimates that are in very good agreement with the PG results.

**Figure 10:**
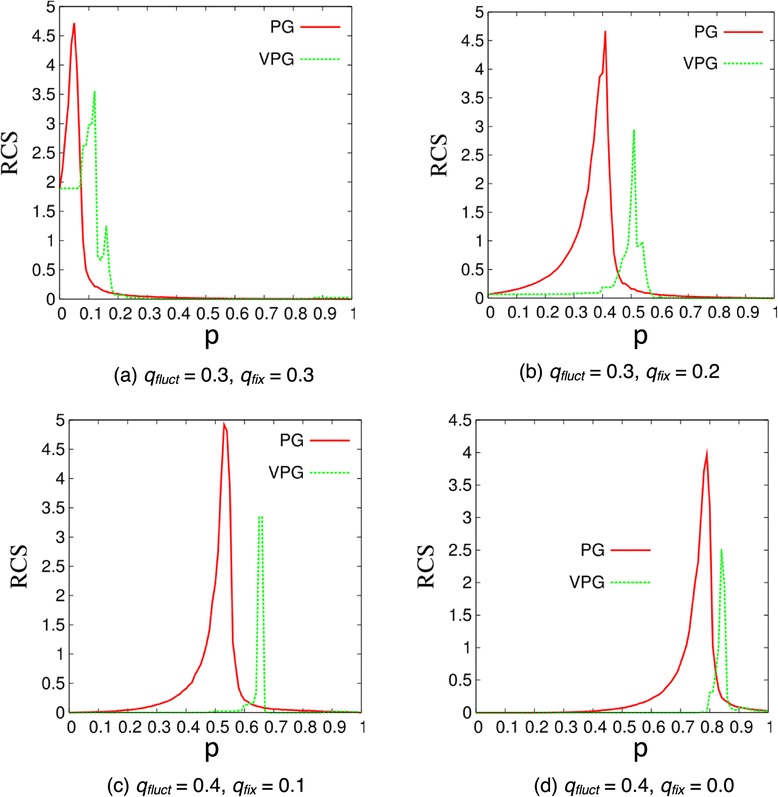
**Rigid cluster susceptibility (RCS) curve for four exemplar lattice cases as a function of the fluctuating edge probability,** ***p*** **, based on ensemble averaged PG and VPG.**

**Figure 11:**
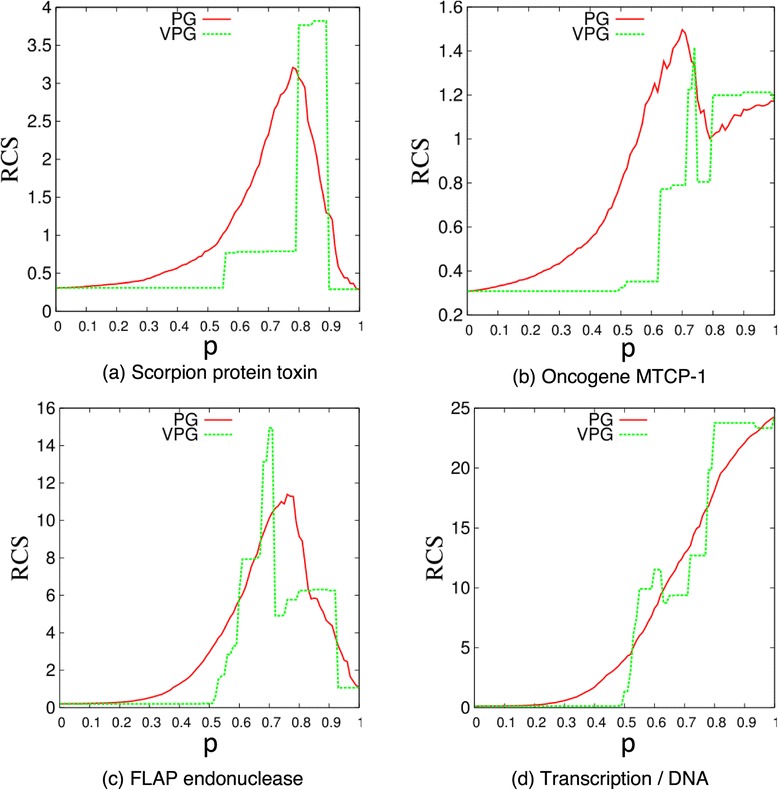
**Rigid cluster susceptibility (RCS) curve for four protein networks as a function of the fluctuating edge probability,** ***p*** **, based on ensemble averaged PG and VPG.** The maximum peak represents the point where the protein transitions from being globally flexible to globally rigid.

The rigid cluster susceptibility (RCS) is compared between the VPG and PG algorithms. The RCS is defined as:

5 $$ RCS=\frac{1}{N_c-1}{\displaystyle \sum_{c=1}^{N_c-1}{\left({N}_V(c)- AC{S}_{reduced}\right)}^2} $$

where the sum in Equation 5 does not include the largest rigid cluster in the network, and the reduced ACS is the average cluster size without counting the largest rigid cluster in the network. The RCS is called the reduced second moment in rigid cluster size in percolation theory, and it is employed to identify the percolation threshold [40]. With this definition, the peak in the RCS as a function of *p* corresponds to a critical value where the rigid cluster size exhibits maximum fluctuations. Typically this point indicates a transition from a globally flexible to a globally rigid network, which is referred to as a rigidity transition [13]. The RCS curves for the exemplar disordered-lattices and four proteins are shown in Figure 10 and Figure 11, respectively. In general the nature of the rigidity transition for these networks is captured well by the VPG, albeit the location of where the transition takes place is inaccurate due to the mean-field nature of the calculation. In all the metrics considered here, the VPG better represents the ensemble-average properties of the PG in proteins compared to the disordered lattices.

We have in prior work compared the detailed rigid cluster decompositions between the VPG and PG to a large dataset of 273 proteins [29]. We found that the correspondence between the average rigid cluster decomposition of the PG to that predicted by the VPG is in markedly good agreement when using a uniform probability, *p*, for H-bonds to be present in the protein. To test this correspondence further, we also developed a hybrid model that bridges the averaging technique of PG with the VPG in a linear weighted fashion so a gradual transition from one model to the other can be analyzed [30]. The hybrid model was able to determine that the VPG provides a very good tradeoff between simplicity, speed, and accuracy that is useful for pragmatic applications. In other work to be published elsewhere, the VPG is applied to proteins when non-uniform probability is used for fluctuating H-bonds given by a Fermi-Dirac probability distribution function [23] where most H-bonds (>80%) have a probability to be formed that is close to either 0 or 1. As such, the distinction between the VPG and the ensemble PG significantly reduces compared to using a constant *p* for all H-bonds as done here. While our other (unpublished) results extend the work presented here in a specialized application, verifying the validity of the VPG through extensive testing demonstrates that VPG is an excellent MFA under conditions that are far worse than what will be encountered in applications to proteins and other biopolymers.

**Figure 12:**
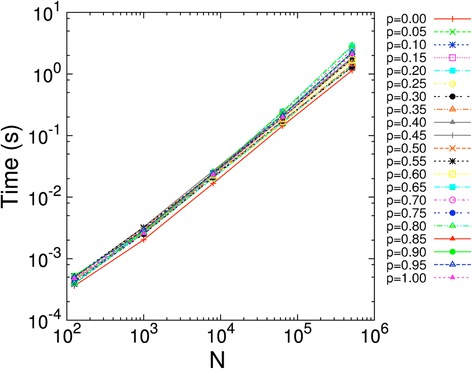
**VPG execution times are plotted versus the number of vertices** ***N*** **within the network, for 21 uniformly spaced values of** ***p*** **for** ***q***_**fix**_ **= 0** **and** ***q***_**fluct**_ **= 1.** In all cases, the execution time is essentially linear with respect to *N*.

The last aspect of the VPG to characterize is the execution time compared to a single PG. As demonstrated in Figure 12, the execution time of the VPG scales approximately linearly with the number of vertices in the network. Typically, one run of the VPG is about 20% faster than a single PG. The accuracy of the VPG algorithm is comparable to the ensemble average over hundreds of PG network realizations in proteins. Thus, the VPG will provide a tremendous increase in computational speed of rigidity analyses in applications involving protein flexibility and stability.

The benchmarking of linear computational complexity holds up for all regions of parameter space {*q*_fix_, *q*_fluct_, *p*}, except *exceedingly* close to the rigidity transition when *q*_fix_ → 0 and *q*_fluct_ → 1, where the VPG scales as *O*(*N*^2^) to identify all constraints as independent or redundant. It is generally found from simulation on networks that model molecular structure that condensation of Laman subgraphs improves the scaling of the VPG from *O*(*N*^2^) to almost always *O*(*N*) above the rigidity transition. This dramatic improvement in performance follows the same response as that found in the 2D and 3D PG’s. Here, “almost always” means that as long as the network is not very near the rigidity transition, we empirically find a linear dependence on run time to the number of vertices in the network. Interestingly, for the case of a complete lattice with all fluctuating edges (*q*_fix_ = 0, *q*_fluct_ = 1, 0 ≤ *p* ≤ 1) the prediction of the rigidity threshold is in perfect agreement with the MCC prediction where the critical transition probability is given by the value of *p* in Equation 1 when one sets *F*_*MCC*_ = 0. Although VPG recovers MCC when no constraint density fluctuations are present, in this atypical case VPG is found to perform as *O*(*N*^2^) very near the rigidity transition, where *p* must be well within a 1% deviation from the threshold probability. However, this worse case situation will never happen in practice when studying molecular systems of any type.

## Conclusions

The algorithm of the VPG has been defined, and the accuracy and performance of a VPG implementation has been benchmarked on a diverse set of networks comprising random disordered lattice networks and four representative proteins. To quantify accuracy, the VPG algorithm is compared to ensemble average properties of the PG algorithm for the number of internal degrees of freedom, average cluster size, and rigid cluster susceptibility. Its accuracy for counting the number of DOF is significantly improved over MCC. The computational cost for the VPG in practice is anywhere between 100 to 1000 times faster than PG ensemble averaging, and no statistical error bars are incurred because the need for sampling over different constraint topologies has been completely eliminated. Moreover, the average Rigid Cluster Size and Rigid Cluster Susceptibility (RCS) compare well between the PG and VPG approaches, whereas the commonly invoked MCC cannot provide this information.

Unlike MCC that averages globally across a network, the VPG is a MFA to the PG algorithm at the level of individual edges. Because of its mean-field nature, the VPG deviates further from the ensemble averaged PG results when distance constraint fluctuations are large and cooperative, and while not necessary, errors are often further exacerbated with increased heterogeneous constraint density fluctuations. Disordered lattices with 3D and 2D topologies provide a convenient way to probe extreme bad cases where the VPG has maximum errors. It is found that the maximum error cases that disordered lattices present are more than an order of magnitude higher than the maximum errors encounter in proteins. Even in the worst case situations found for disordered lattices, the VPG provides a faithful qualitative representation of the exact ensemble averaged PG results that provides far more information than the commonly employed MCC.

In proteins the VPG provides a quantitatively accurate estimate of the average mechanical properties of the PG results over an ensemble of hundreds of samples. Because the VPG performs about 20% faster than one PG run, it provides a pragmatic alternative to averaging PG rigidity characteristics over an ensemble of constraint topologies. It is clear that the utility of the VPG falls in between the most accurate but slowest method of ensemble averaging over hundreds to thousands of independent PG runs, and the fastest but least accurate MCC. The VPG promises to offer the computational biology community a powerful tool for the mechanical analysis of protein networks suitable for high-throughput applications in structural bioinformatics.

## Appendix

In this appendix, descriptions and pseudocodes are provided for key components to the VPG. The pseudocodes are written without reference to data structure details that represent the network.

### Description of the key VPG algorithms

*virtural_pebble game(edge_list, N)* considers a list of edges to be place among N vertices, and tries to cover each edge with as many pebbles as its capacity one at a time. To cover an edge, first pull back 6 pebbles associated with one of the incident vertices of the edge. Rigidity theory guarantees that 6 pebbles can be pulled back for any vertex. Then a second pebble search is carried out for the second incident vertex, while blocking access to the first vertex. If enough pebbles can be found to cover the edge, the search is successful; otherwise the search fails and all the vertices that are visited during the search condensate to one representative vertex. The same procedure is repeated for all the edges.

*get_pebbles(u, pebbles_required, block_vertex)* attempts to retrieve the required number of free pebbles on vertex u, and it returns the number of free pebbles on vertex u after the attempt is made. Two possible outcomes are possible: Case 1: The number of free pebbles residing on vertex u is greater than or equal to the required number of pebbles. Case 2: The maximum number of pebbles is on vertex u, but it is less than the required number. The visited vertices are also given as output, but in case 2, no other vertex can be reached by a pebble search, meaning this list of vertices define a Laman subgraph.

*collect_pebbles(v, visited_vertices, pebbles_required)* performs multiple breadth first searches to find free pebbles and pulls them onto the root vertex v of the search tree by reversing the search route that leads from vertex v to the free pebbles. Pebble searching continues until either the required pebbles are collected on vertex v, or the required number of pebbles is more than can be collected.

*back_track(v, vertex_front, route)* follows in reverse the route previously taken that found free pebbles at the last vertex_front and it pulls free pebbles through a non-bifurcating chain of vertices back to vertex v while covering the edges as a new directed path. A multiple set of created directed paths start from on the vertex_front and traverse to the root vertex, v. The number of pebbles that can be pulled back is limited to the sum over minimum edge capacities encountered along each backward track.

*collapse_vertices(visited_vertices)* is used following a failed pebble search. This algorithm chooses the vertex with lowest index as root, and all vertices in the visited vertices are assigned the same index as the root index. Hence all the vertices in the visited_vertices list are grouped together and collapsed onto one representative vertex – the root. Any edges that are remaining to be placed in the network, but have both of its incident vertices belonging to this collapsed set of vertices are removed from the edge_list.

### VPG pseudocodes

Remark: With straightforward data structures the presented algorithms perform as O(N^2^). Our implemented data structure employs a recursive mapping functionality such that when a vertex is needed, a dynamic linked list (DLL) interface is always called to return the lowest vertex label. The DLL is updated (shrinks) among active vertices upon its use, but most vertices are not active and not updated. The DLL interface is not shown because it obfuscates the procedural steps in the above five algorithms, but it improves typical performance to O(N) as explained in the text.
